# Successful pocket creation method using a multimodal device in endoscopic submucosal dissection for a colonic lateral spreading tumor

**DOI:** 10.1055/a-2852-7351

**Published:** 2026-05-20

**Authors:** Mitsuru Esaki, Norio Fukami, Daisuke Yamaguchi, Ibrahim H. Hassan, Hyun Jae Kim, Terry L. Jue

**Affiliations:** 1 Division of Gastroenterology and Hepatology 23387 Mayo Clinic ArizonaScottsdale, ArizonaUnited States; 2 Department of Medicine and Bioregulatory Science, Graduate School of Medical Sciences 12923 Kyushu UniversityFukuokaJapan; 3 Division of Gastroenterology, Department of Internal Medicine, Faculty of Medicine 13030 Saga UniversitySagaJapan

**Fig. 1:**
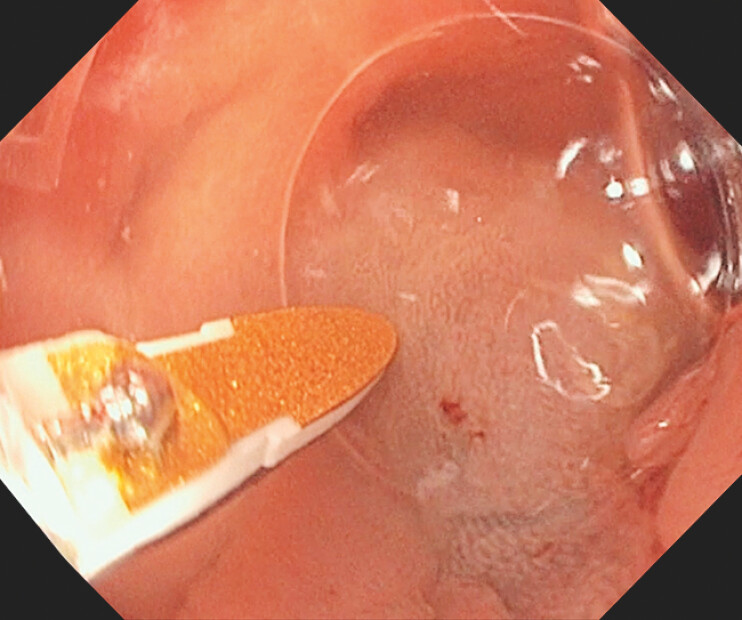
The image of the Speedboat UltraSlim device.

The pocket creation method (PCM) in endoscopic submucosal dissection (ESD) maintains a consistent dissection plane parallel to the muscularis propria, reducing the risk of intraoperative perforation 1 2 . However, intraoperative bleeding within the pocket can obscure visualization and complicate the procedure 3 . The Speedboat UltraSlim (SpB-US; Creo Medical Ltd, UK, Fig. 1 ) is a multimodal device integrating bipolar radiofrequency cutting (BRC), microwave coagulation (MWC), and a through-the-needle injection function 4 5 . This case report demonstrates the successful application of PCM using SpB-US in ESD for a colonic neoplasm with excellent bleeding prevention and control.

**Fig. 2:**
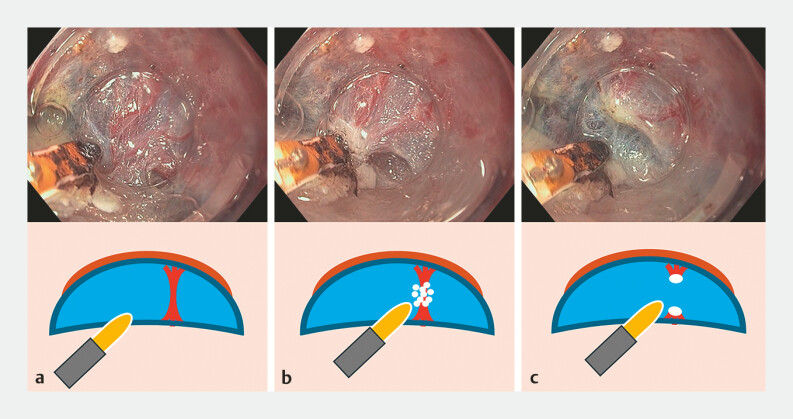
The pocket creation method using the Speedboat UltraSlim device in colorectal endoscopic submucosal dissection. **a** Large vessels exposed in the pocket. **b** Pre-coagulation of large vessels with gentle compression using micro-wave coagulation. **c** Effective vessel sealing and cutting without significant bleeding.

A 50 mm lateral spreading tumor in the rectum treated by ESD with PCM using SpB-US ( Video 1 ). After injection beneath the lesion, BRC at 30 W was applied for the initial mucosal incision on the anal side to create a mucosal flap for submucosal entry. MWC at 9W was applied for hemostasis for intraoperative bleeding. During the submucosal dissection within the pocket, several large vessels were encountered ( Fig. 2 **a** ). After precise trimming of the surrounding submucosal fibers, vessels were pre-coagulated with gentle compression using MWC at 9W for approximately 5 seconds. Effective vessel sealing and cutting were achieved without significant bleeding ( Fig. 2 **b, c** ). The insulated hull of the device was kept onto the muscular layer to avoid thermal injury by the rotation of SpB-US. En bloc resection was achieved in 64 minutes without complications and the need for hemostatic forceps. Histopathology revealed a tubule-villous adenoma with negative margins.

The successful pocket creation method using a Speedboat UltraSlim device in endoscopic submucosal dissection for a colonic lateral spreading tumor.Video 1

MWC demonstrated effective vessel management and bleeding control during ESD. MWC provides more gradual and uniform coagulation effects than conventional monopolar current. Additional coagulation can be applied on demand if oozing of blood is noted. A seamless transition to cutting is possible. The combination of PCM with MWC of SpB-US appears to be a valuable option for ESD.

Endoscopy_UCTN_Code_TTT_1AQ_2AD_3AD
